# Traditional Chinese Medicine Da-Cheng-Qi-Tang Ameliorates Impaired Gastrointestinal Motility and Intestinal Inflammatory Response in a Mouse Model of Postoperative Ileus

**DOI:** 10.1155/2020/9074069

**Published:** 2020-07-31

**Authors:** Chunqiu Chen, Min Li, Xiaohong Liu, Jianwei Fan, Hong Zhang, Sisi Lin, Lu Yin, Jakub Fichna, Yongyu Li

**Affiliations:** ^1^Department of General Surgery, Shanghai Tenth People's Hospital, Tongji University School of Medicine, Shanghai 200072, China; ^2^Institute of Digestive Disease, Tongji University School of Medicine, Shanghai 200092, China; ^3^Department of Physiology, Zunyi Medical College, Zunyi 563000, China; ^4^Medical Experiment Center, Shaanxi University of Chinese Medicine, Xianyang, Shaanxi 712046, China; ^5^Department of Biochemistry, Faculty of Medicine, Medical University of Lodz, Lodz, Poland

## Abstract

This study was to explore the therapeutic effect and mechanism of the traditional Chinese medicine with the formula Da-Cheng-Qi-Tang (T-DCQT) and a modified Da-Cheng-Qi-Tang (M-DCQT) in a postoperative ileus (POI) mouse model. POI was induced via small bowel manipulation, and T-DCQT or M-DCQT was given by enema. The intestinal motility was measured with a charcoal mixture gavage. The intestinal tissues were collected for further studies by histopathological, qPCR, immunohistochemical staining, and Western blotting. Levels of inflammatory cytokines in blood were determined using a high-throughput liquid chip. We found that gastrointestinal dysfunction was alleviated after administration of either a T-DCQT or M-DCQT enema. Increased expression of NF-*κ*B, p38 MAPK, and TLR4 in the intestinal tissues of POI mice were reversed following treatment. IL-1*α*, IL-6, MIP-1*β,* and IL-17 levels were significantly reduced at 24 h and 48 h following treatment, while the MCP-1 level was only observed to be reduced at 24 h after the treatment. Furthermore, compared with the T-DCQT effect, M-DCQT treatment was more effective in alleviating the increased IL-6, MIP-1*β,* and IL-1*α* levels. So, we draw a conclusion that T-DCQT or M-DCOT could ameliorate the POI-associated inflammatory response and improve GI motility in a POI mouse model.

## 1. Introduction

Patients who undergo an abdominal surgical procedure will develop a transient episode of gastrointestinal (GI) dysfunction, even when minimally invasive approaches are used. Some of these patients will go on to develop a more serious GI motility disorder, namely postoperative ileus (POI). Development of POI in combination with gut inflammation can lead to impaired motility of the entire GI tract [1], which negatively impacts patient morbidity and prolongs hospital stays [2]. The mechanism that causes impaired I motility in the context of POI is likely multifactorial, with inflammatory cell activation, autonomic dysfunction, modulation of the GI hormone activity, and electrolyte imbalance all playing a role [3]. Therapies for treating POI include prokinetics, opioid antagonists (alvimopan), and ghrelin agonists. Although most of the existing therapies are effective in shortening the duration of POI, a Cochrane review indicates that routine administration of many established prokinetics (metoclopramide, cisapride, erythromycin, cholecystokinin, and dopamine antagonists) is not recommended for POI prevention [4]. Many of the existing therapies also have undesirable side effects and high associated costs [5]. Therefore, there is still a need for more effective and economical POI therapies.

The traditional Chinese formula Da-Cheng-Qi-Tang (T-DCQT) is composed of 4 Chinese medical herbs, *Rheum palmatum* L. (Dahuang), *Magnolia henryi* Dunn. (Houpu), *Citrus aurantium* L. (Zhishi), and *Natrii sulfas* (Mangxiao). In China, T-DCQT decoctions have been used to effectively manage a variety of digestive diseases, including ileus, for many years [6, 7]. T-DCQT is reported to promote GI motility by protecting the enteric nervous system (ENS), upregulating the expression of several neurotransmitters (ACh, SP, VIP, and NOS) [8], and lowering the levels of proinflammatory cytokines in pancreatitis-associated intestinal dysmotility [9]. The present study investigated whether T-DCQT could ameliorate impaired GI transit and intestinal inflammation of POI. In addition, given that Chinese angelica, ginseng, *Rhizoma Atractylodis macrocephalae,* and *Radix Paeoniae Alba* might benefit the recovery of the body from surgery strike by improving the circulation and immune function according to traditional Chinese medicine theory, they, therefore, were added to T-DCQT as a modified Da-Cheng-Qi-Tang (M-DCQT) for experimental treatment in the study also. The efficacy and mechanism of the T-DCQT and M-DCQT were then evaluated in a POI mouse model.

## 2. Materials and Methods

### 2.1. Animal Studies

Adult male and female Kunming mice (*n* = 8 per group, weighing 20–22 g) were obtained from the Experimental Animal Center of the Air Force Military Medical University, China. Mice were housed in the experimental animal center in controlled, pathogen-free conditions and kept at a constant temperature (22°C) with 12 : 12-hour light-dark cycle and free access to standard laboratory tap water. All animal study protocols were developed according to the International Guidelines for Care and Use of Laboratory Animals and were approved by the Animal Ethics Committee of the Shaanxi University of Chinese Medicine. All procedures were strictly in accordance with “The Guide for Care and Use of Laboratory Animals” issued by Shaanxi University of Traditional Chinese Medicine (SUCMLAEC 2018008).

POI was induced using a small bowel manipulation method as previously reported [10, 11]. In brief, mice were anesthetized via intraperitoneal injection of 2% chloral hydrate at a dose of 0.15 ml/10 g, and the abdomen was shaved and sterilized with 75% ethanol. A 1 cm midline abdominal incision was made so that the small intestine from the ligament of Treitz to the terminal ileum could be carefully externalized onto wet gauze using two saline-moistened cotton swabs. Then, this section of the small intestine was systematically touched with two saline-moistened cotton swabs for 5 minutes, and this was repeated 3 times. The sham treatment group only underwent the laparotomy procedure without any bowel manipulation.

After suturing the abdomen, mice were randomly assigned to receive either T-DCQT, M-DCQT, or normal saline solution (N. S) treatment.

### 2.2. Evaluation of Intestinal Motility

Intestinal motility was measured 24 hours or 48 hours after surgery according to the previous experimental method [10]. Briefly, mice were treated with a 5% charcoal marking mixture by oral gavage (0.1 mL per 10 g body weight), 0.5 g charcoal + 0.25 g gum arabic in 10 ml normal saline. Both charcoal and gum arabic were purchased from Tianjin Kermel Reagent Co. After 20 minutes, mice were sacrificed via enflurane inhalation, and the whole small intestine was taken out immediately. The total length of the small intestine and the pushing length of charcoal marking mixture in the small intestine were measured, and the intestinal motility was expressed as small intestinal transit (%) = the charcoal pass distance/the total length of the small intestine ×100. Then, the propulsion rate of mice in different groups was compared with that of normal control which was calculated as 100%.

### 2.3. Sample Collection and Detection

At 24 h and 48 h after operation, mice were reanesthetized, and the ileum was removed. Blood was exsanguinated from the inferior vena cava. Blood samples were centrifuged at 4000 g for 10 min at 4°C. The ileum tissue and plasma samples were then stored at −80°C for subsequent experiments.

#### 2.3.1. Evaluation of Pathological Alterations

Intestinal sections were fixed in 10% neutral-buffered formalin, embedded in paraffin, and cut into 5 *μ*m sections on a microtome. The sections were stained with hematoxylin and eosin (H&E). Three specimens from each treatment group and five representative areas from each slide were selected for blinded evaluation of damage of the ileum after surgery/treatment.

Some formalin-fixed, paraffin-embedded sections were used to perform immunohistochemical staining for NF-*κ*B, p38, and TLR4. In brief, tissue sections were incubated with either a mouse NF-*κ*B primary antibody (SC-8008), a rabbit p38 primary antibody (SC-535), or a mouse TLR4 primary antibody (SC-293072) (1 : 100; Santa Cruz, USA) at 4°C overnight. The sections were then washed in 10 mM phosphate-buffered saline (PBS; pH 7.4) (Thermo Fisher Scientific) at room temperature three times and then incubated with a goat anti-rabbit horseradish peroxidase- (HRP-) conjugated IgG secondary antibody (1 : 10000; Cell Signaling Technology®) at room temperature for 30 min. The sections were then washed 3 times in 10 mM Tris-buffered saline (pH 8.4) for 2 min at room temperature and then washed with 10 mM PBS (pH 7.4) three times at room temperature. The sections were then incubated with 200 *μ*l of 3,3′-diaminobenzidine substrate (Thermo Fisher Scientific) for 5 min at room temperature, followed by washing with distilled water at room temperature for 5 min.

Following protein staining, counterstaining and scoring were performed by a pathologist. The level of immunostaining in tissue samples was evaluated using a standard scoring system based on the proportion of positively stained cells and the level of staining intensity.

#### 2.3.2. RNA Extraction and Quantitative Real-Time Polymerase Chain Reaction (qRT-PCR)

The primers used for qPCR studies are listed in Table 1.

Total RNA was isolated from fresh ileum and colon tissues using TRIzol (Invitrogen, New York, NY, USA; Cat. no. 15596–026). The concentration of RNA and absorbance ratio for 260 nm/280 nm (OD260/280) were verified using a NanoDrop ND-1000 Spectrophotometer (Thermo Fisher Scientific, Waltham, MA, USA). After DNase I treatment, the RNA was transcribed into cDNA using a Prime Script RT Reagent Kit (Takara, Tokyo, Japan; Cat. no. RR037A) according to the manufacturer's instructions in an Eppendorf Mastercycler Personal Thermo Cycler with the following method: 37°C for 15 min, followed by 85°C for 5 s, and then cooling at 4°C. qRT-PCR was conducted in a total reaction volume of 20 *μ*L with SYBR Premix EX Taq II (Takara, Tokyo, Japan; Cat. no. RR820A) with the following method: 30 seconds at 95°C, followed by 40 cycles of 5 seconds each at 95°C and 31 seconds at 60°C. Melt curve analysis was performed to confirm the specificity of the qRT-PCR. qRT-PCR studies were performed with all samples in triplicate, and the results were normalized to the expression of glyceraldehyde 3-phosphate dehydrogenase (GAPDH) and then analyzed with the 2^−ΔΔCt method.

#### 2.3.3. Protein Preparation and Western Blotting

According to previously described methods, ileal tissue segments were homogenized and lysed with ice-cold 1 × RIPA buffer containing inhibitors. The protein concentration was determined using the BCA method. The supernatant was isolated and mixed with Laemmli buffer and then boiled for 10 minutes at 95°C. Equal amounts of protein were loaded into 10% gels to be separated by sodium dodecyl sulfate-polyacrylamide gel electrophoresis (SDS-PAGE). After separation by SDS-PAGE, the protein in the gel was transferred to a polyvinylidene difluoride (PVDF) membrane. Following transfer, the membrane was blocked for 2 h with 5% nonfat milk in TBS to minimize binding of nonspecific antigens. The membranes were then washed with TBS and incubated with primary antibody overnight at 4°C.

Primary antibodies used included anti-NF-*κ*B, TLR4, p-p38, and p38 MAPK. After incubation in primary antibody, the membranes were washed in TBS and incubated with horseradish peroxidase-conjugated secondary antibodies for 1 h at room temperature. The protein bands were detected using an ECL chemiluminescent detection system. The densities of target protein bands were then normalized to that of *β*-actin. Western blot analysis was conducted with Image studio version 4.0.

#### 2.3.4. Liquid Chip Method to Detect the Expression of Inflammatory Cytokines

The expression levels of the plasma cytokines IL-1*α*, IL-6, MCP-1, MIP-1*α*, MIP-1*β*, IL-17, INF-*γ*, IL-4, IL-10, IL-13, and TNF-*α* were determined using Luminex technology (MILLIPLEX MAP Human Cytokine/Chemokine Panel; Cat no: HCYTOMAG-60K, Millipore, St. Charles, MO). This bead-based assay utilizes fluorescent color-coded beads precoated with capture antibodies that target specific cytokines. Plasma samples were filtered through 0.22 *μ*m spin filters and run in duplicates for each assay. Duplicates did not vary by more than 5%. Samples were assayed using Luminex 200 (USA) [12].

### 2.4. Drugs

T- and M-DCQT composed of spray-dried medicinal herbs (crude drug) are listed in Table 2, and they were purchased from Shaanxi Traditional Chinese Medicine Pieces Co., Ltd. (Shaanxi, China). The crude formula components were extracted, concentrated, and used as follows.

According to the manufacturer, the T-DCQT decoction was prepared in the standard ratio of 12 : 15 : 12 : 9. One dose of the mixture was steeped in cold water for 2 h. After decocting the mixture twice (first decoction was 90 min long, and second decoction was 60 min long; *Rheum palmatum* L. was added 70 min late into the first decoction period), the T-DCQT decoction was mixed and filtered. 48 g of crude drug in sterile distilled water was concentrated into 96 ml to generate a 0.5 g/ml solution. Taking into account the effective dose of T-DCQT in patients and the difference in body surface area between human and animals, T-DCQT was administered to mice at a dose of 0.1 ml/10 g body weight. The procedure for preparing the M-DCQT decoction was similar to the procedure used to prepare T-DCQT.

The mice in the T-DCQT and M-DCQT groups received their respective drug decoction via transanal enema at a dose of 0.1 ml/10 g body weight, twice per day (at 8 hours interval). The drug enema is administered by inserting 2 cm into the rectum and is kept in place for 15 min. In other experimental groups, normal saline was delivered by transanal enema.

### 2.5. Experimental Design

In pilot experiments, mice were euthanized at 4, 12, 24, and 48 h after operation (*n* = 8–10 per group). Based on the previous experiments, we selected 24 h and 48 h as the designated time points for all subsequent experiments and analyses.

Mice were randomly divided into the experimental groups, which were the control group, sham group, POI group, T-DCQT group, and M-DCQT group. Mice in each experimental group were also randomly divided into 24 h or 48 h experimental subgroups (*n* = 8 per subgroup).

### 2.6. Data Analysis

Results are expressed as the mean ± standard error of the mean (SEM) or mean ± standard deviation (SD), which are indicated in the figure legends. Statistical analysis was performed using IBM SPSS statistics 19. Data quality was verified with Levene's test for equality of variances. The results were then analyzed with one-way ANOVA, followed by the least-significance difference (LSD) test. Some results were also analyzed using Kruskal–Wallis and Mann–Whitney *U* tests. Differences with *p* < 0.05 were considered statistically significant.

## 3. Results

### 3.1. Intestinal Motility Impairment in POI Mice is Alleviated by T-DCQT or M-DCQT Treatment

**Figure 1:**
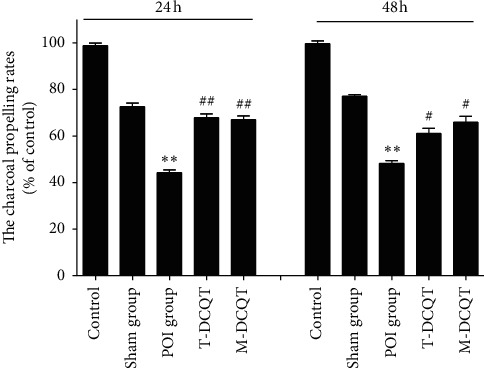
GI transit rates in different treatment groups at 24 h and 48 h after intestinal manipulation. Data are shown as mean ± SD (*n* = 6), ^*∗∗*^*p* < 0.01 vs. the sham group and ^#^*p* < 0.05 and ^##^*p* < 0.01 vs. the POI group.

The percentage of carbon powder passed through the small intestine was used as a read out of intestinal motility. As shown in Figure 1, in control and sham treatment groups, no significant differences in the transit rate were observed after 24 h or 48 h (*p* > 0.05). However, the intestinal transit rate was significantly reduced in mice that underwent intestinal manipulation (POI group) at both 24 h and 48 h after intestinal manipulation (*p* < 0.01). Impaired GI motility was partially ameliorated in the groups of mice that received a T-DCQT or M-DCQT enema treatment. The T-DCQT and m-DCQT treatment groups displayed a significantly faster intestinal transit rate than that of the POI group (*p* < 0.01) at 24 h and (*p* < 0.05) at 48 h following intestinal manipulation.

### 3.2. POI-Associated Histological Changes in the Ileum are Improved in POI Mice Treated with T-DCQT or M-DCQT

Following intestinal manipulation, the ileum of POI mice showed significant histopathological alterations, including increased numbers of mucosal lymphocytes, villous epithelial cell structural damage, epithelial cell necrosis, submucosal edema, hyperemia, and inflammatory cell infiltration. However, in the control group, no significant histopathological changes were observed, except the presence of neatly arranged villi in the intestinal wall and mucosa integrity.

**Figure 2:**
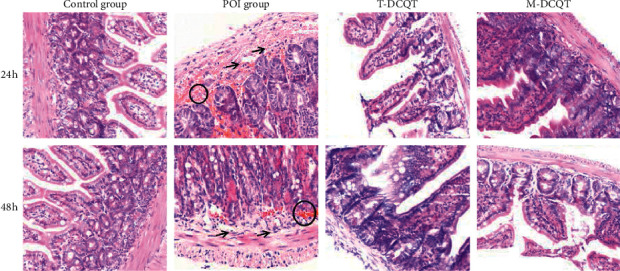
Pathological changes in the ileum. Representative micrographs of hematoxylin and eosin- (H&E-) stained tissues at 24 h and 48 h after intestinal manipulation (original magnification 200x). Hyperemia is indicated with a circle O, and inflammatory cells infiltration is indicated with arrows (⟶).

Compared with the POI group, the ileum of the T-DCQT and M-DCQT treatment groups displayed significantly less mucosal injury, mild epithelial cell swelling, and diminished inflammatory cell infiltration (Figure 2).

### 3.3. mRNA Level of NF-*κ*Bp65 and p38 MAPK in the Ileum of Different Groups

Analyzing NF-*κ*Bp65 and p38 MAPK expressions by qPCR revealed that the mRNA levels of both were significantly increased in the POI group 24 h after intestinal manipulation when compared with the control group (*p* < 0.05).

**Figure 3:**
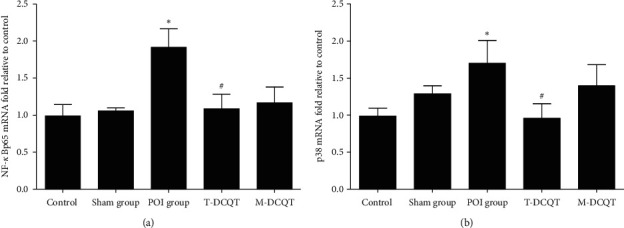
(a). NF-*κ*Bp65. (b) p38 MAPK mRNA expression in the ileum in different treatment groups at 24 h after intestinal manipulation by qRT-PCR (mean ± SEM, *n* = 4). ^*∗*^*p* < 0.05 vs. the control group and ^#^*p* < 0.05 vs. the POI group.

NF-*κ*Bp65 and p38 MAPK mRNA expressions were lower in the ileum of the T-DCQT and M-DCQT treatment groups when compared to the POI group, with expression more obviously decreased in the T-DCQT treatment group (*p* < 0.05) (Figures 3(a) and 3(b)).

### 3.4. Changes in NF-*κ*B, p38 MAPK, and TLR4 Expression in the Ileum Detected via Immunohistochemistry

**Figure 4:**
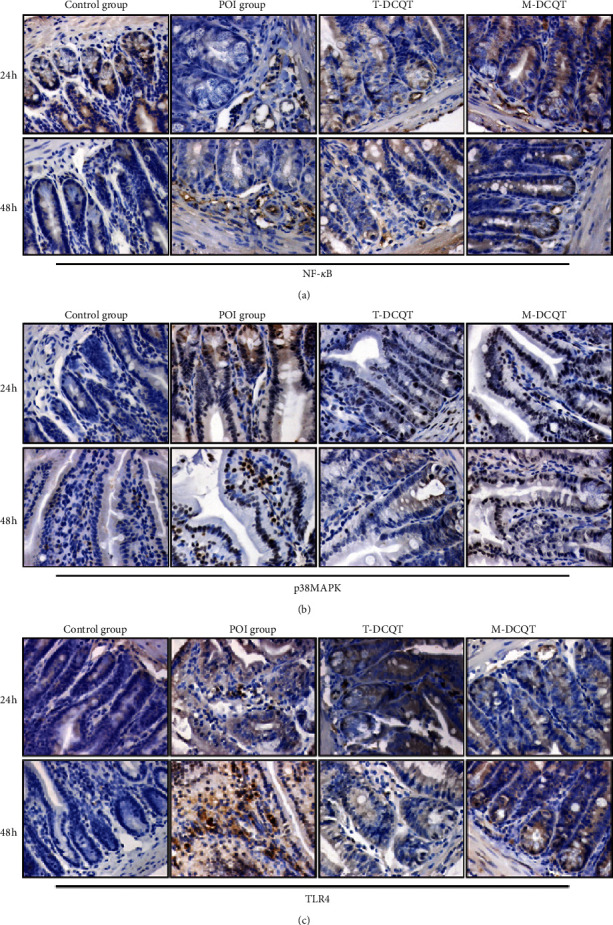
Expression of (a) NF-*κ*B, (b) p38 MAPK, and (c) TLR4 in the ileum of different groups after intestinal manipulation at 24 h and 48 h (IHC × 400) via immunohistochemistry.

Immunohistochemical staining showed that no clear expression of NF-*κ*B, p38 MAPK, or TLR4 was observed in the ileum of the control group. The staining for NF-*κ*B, p38 MAPK, and TLR4 in the ileum of the POI group was positive and significantly increased at 24 h and 48 h following intestinal manipulation. The expression of these proteins was mainly concentrated in the area of the intestinal mucosa and submucosa where inflammatory cell infiltration occurs. In contrast, intestinal tissues from the T-DCQT and M-DCQT treatment groups showed significantly diminished positive staining for NF-*κ*B, p38 MAPK, and TLR4 (Figures 4(a)–4(c)).

### 3.5. The Expression of NF-*κ*B, p38 MAPK, p-p38, and TLR4 in the Small Intestine of POI Mice Detected by Western Blot

**Figure 5:**
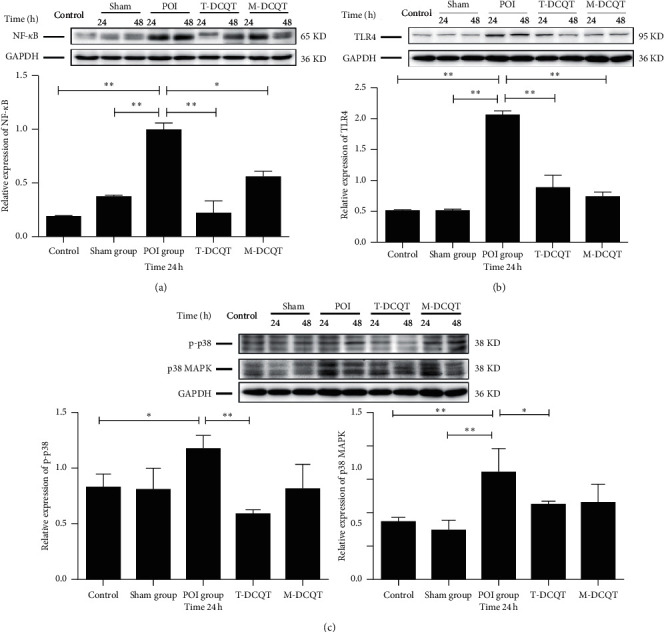
The protein expression of (a) NF-*κ*B, (b) TLR4, and (c) p-p38 and p38 MAPK in the ileum of different groups after intestinal manipulation at 24 h and 48 h determined via Western blot. Compared with different groups at 24 h (mean ± SEM, *n* = 3), ^*∗*^*p* < 0.05 and ^*∗∗*^*p* < 0.01.

As shown in Figures 5(a)–5(c), the expression levels of the NF-*κ*B, TLR4, p38 MAPK, and its phosphorylation form p-p38 proteins in the intestinal tissue (ileum) were determined via Western blot. The expression of NF-*κ*B (p65), TLR4, p38 MAPK, and p-p38 in the POI group was significantly increased after intestinal manipulation in 24 h and 48 h when compared to that in the control group or in the sham group. To some extent, NF-*κ*B, p38, p-p38, and TLR4 expression levels were suppressed in both the T-DCQT and M-DCQT treatment groups. The semiquantitative data of 24 h in each group were statistically analyzed. When compared with that in the POI group, NF-*κ*B expression was significantly decreased in both T-DCQT and M-DCQT treatment groups (*p* < 0.05 or *p* < 0.01) (Figure 5(a)), and TLR4 expression was significantly decreased too (*p* < 0.01) (Figure 5(b)). Furthermore, p38 MAPK and p-p38 expressions were significantly decreased in the T-DCQT group when compared with that in the POI group (*p* < 0.05 or *p* < 0.01) (Figure 5(c)); however, the inhibitory effect of M-DCQT was weak (*p* > 0.05) (Figure 5(c)).

### 3.6. Serum Cytokines Level in POI Mice Detected by the Liquid Chip Method

**Figure 6:**
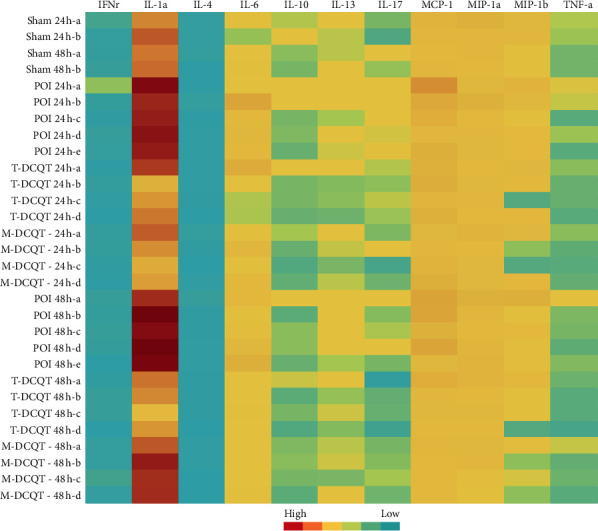
Cytokine expression in serum of different treatment groups at 24 h and 48 h after intestinal manipulation (original figure of the high-throughput liquid chip, partial representation).

**Figure 7:**
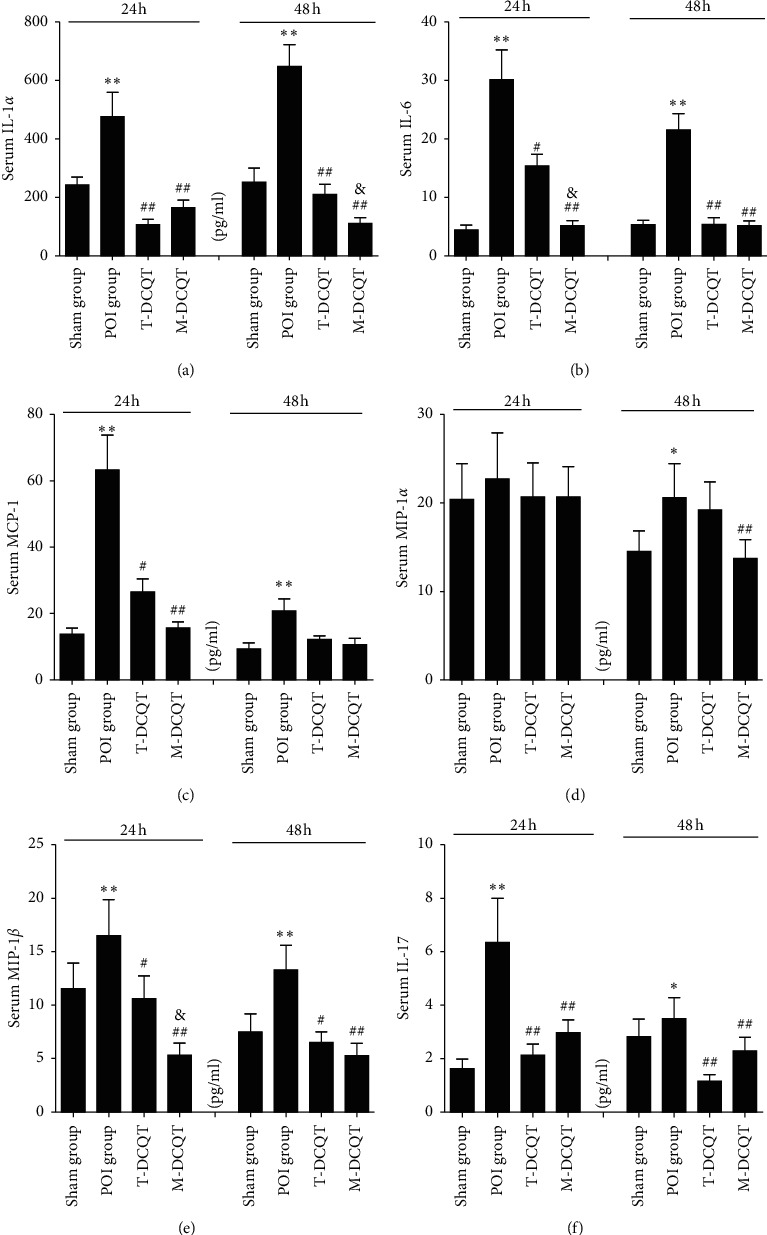
The inflammatory cytokines serum expression (a) IL-1*α*, (b) IL-6, (c) MCP-1, (d) MIP-1*α,* (e) MIP-1*β,* and (f) IL-17 in different groups at 24  h and 48 h after intestinal manipulation by a liquid chip (mean ± SEM, *n* = 5); ^*∗∗*^*p* < 0.01 vs. the sham operation group; ^##^*p* < 0.01 *vs*. the POI group; and ^&^*p* < 0.05 vs T-DCQT.

A high-throughput liquid chip method was used to detect the expression of cytokines in mice serum in 24 h and 48 h after intestinal manipulation. It was shown in the representative map (heat map) (Figure 6) and statistical analysis chart (Figures 7(a)–7(f)) that the serum levels of some cytokines changed significantly. Compared with the sham group, the expression levels of the cytokines, such as IL-1*α*, IL-6, MCP-1, MIP-1*α*, MIP-1*β,* and IL-17, in the POI group were significantly increased at 24 h and 48 h after operation (*p* < 0.05 or *p* < 0.01), but the level of MIP-1*α* increased significantly only in the 48 h after operation (*p* < 0.05). T-DCQT or M-DCQT could significantly reduce the expression of these inflammatory cytokines (*p* < 0.05 or *p* < 0.01), but MCP-1 level decreased significantly only in 24 h of operation. In addition, compared with the T-DCQT group, IL-1*α* levels decreased more in 48 h (Figure 7(a)), and IL-6 and MIP-1*β* levels decreased more significantly in 24 h time point (*p* < 0.05) (Figures 7(b) and 7(e)) in the M-DCQT group. There were no significant changes in the serum levels of some other cytokines such as INF-*γ*, IL-4, IL-10, IL-13, and TNF-*α* after operation.

## 4. Discussion

POI is an iatrogenic disorder characterized by a temporary disturbance in gastric and bowel motility following surgery. Its pathophysiology is complex and involves pharmacological (opioids and anesthetics), neurohumoral, and immune-mediated mechanisms. POI increases the risk of postoperative complications and morbidity [3]. Recent studies have shown that postoperative inflammatory reactions are involved in the development of POI [10, 13, 14]. Consistently, with earlier reports [6, 7] at 24 h and 48 h after intestinal manipulation, we observed a significant decrease in the GI transit rate and induction of an inflammatory response characterized by leukocyte infiltration in the intestinal tissue, villous epithelial cell structural damage, submucosal edema, hyperemia, and epithelial cell necrosis.

Inflammatory plays a key role in the pathogenesis of POI. Da-Cheng-Qi-Tang (T-DCQT), a traditional Chinese herbal decoction, is an attractive potential treatment for POI as it is well-known in China for its anti-inflammatory effects. In clinic, it is used in the treatment of acute simple intestinal obstruction, acute cholecystitis, respiratory distress syndrome, crush syndrome, acute appendicitis, and so on. On the other hand, according to traditional Chinese medicine theory, it should be used with caution in cases of old age and weak body, so as to avoid the loss of healthy Qi. And pregnant women should not use it because this prescription is a purgative decoction.

Some contemporary reports [15] have demonstrated that administration of T-DCQT can alleviate acute renal, pancreatic, intestinal, and lung injuries by modulating levels of inflammatory cytokines. For example, in rats with severe acute pancreatitis, treatment with T-DCQT helps regulate the inflammatory response, as it has potent anti-inflammatory and prokinetic actions in this context [16]. Moreover, the herbs Chinese angelica, Ginsen*g, Rhizoma Atractylodis Macrocephalae*, and *Radix Paeoniae Alba* were selected as additions to the traditional DCQT decoction in this study because they can help to promote the recovery of some disturbances after operation, especially the deficiencies in Qi and blood according to traditional Chinese medical theory. For example, Chinese angelica is beneficial for improving blood circulation, and it is also laxative. Ginseng is found in therapies developed for reducing fatigue and enhancing physical performance. *Rhizoma Atractylodis Macrocephalae* is useful for improving appetite and digestive functions, and *Radix Paeoniae Alba* is efficacious in clearing inflammation (heat and dampness in the body in Chinese medicine theory). Therefore, in the study, we investigated the therapeutic effect of T-DCQT and modified Da-Cheng-Qi-Tang (M-DCQT) in a murine POI model and investigated their possible mechanisms of action.

The restoration of normal GI transit is considered to signal patient recovery from POI following surgery. In previous studies, T-DCQT treatment has been shown to help maintain the integrity of enteric nerve-interstitial cells in the context of the Cajal-smooth muscle cell network [17]. T-DCQT is also known to increase plasma motilin [7] and to act as an anti-inflammatory agent [18]. Therefore, it is reasonable that T-DCQT administration may be effective in suppressing intestinal inflammation responses and reversing diminished GI transit. To understand the mechanism by which T-DCQT and M-DCQT treatment promote GI transit, this study examined the inflammatory response following surgery and medical treatment by evaluating local inflammation-related signal pathways and plasma cytokine levels.

It is now generally accepted that the local muscular macrophage population plays a central role in POI pathology [19, 20]. These macrophages are activated with surgical manipulation but are quiescent under normal physiological conditions. Activation of these macrophages results in activation of transcription factors, such as nuclear factor *κ*B (NF-*κ*B), signal transducer and activator of transcription 3 (STAT3), early growth response protein 1 (EGR-1), and production of proinflammatory cytokines, chemokines, integrins, and cell adhesion molecules [21, 22]. Furthermore, macrophages are potently activated by bacterial cell wall molecules (such as LPS), which interact with Toll-like receptors (TLRs) to promote inflammatory responses [23]. As intestinal permeability is transiently increased following intestinal manipulation, bacterial translocation may represent another potential mechanism by which resident macrophages can be stimulated [10, 24]. Manipulation of the intestine triggers the influx of leukocytes in manipulated intestinal segments, impairing the contractile properties of the inflamed intestine [25]. In this study, we found that the expression of NF-*κ*B, p38 MAPK, TLR4, and other components of inflammation-related signaling pathways were significantly increased following intestinal manipulation.

NF-*κ*B has been reported to play a key role in the development of numerous inflammatory diseases. Activation of NF-*κ*B promotes the expression of gene programs that promote transcription of inflammatory cytokines [26]. Moore et al. [27] demonstrated that following colonic manipulation and subsequent impairment of colonic contractility, increased NF-*κ*B protein expression in mouse colon muscularis extracts, increased transcription of genes encoding proinflammatory mediators, and increased inflammatory cell infiltrate were observed. In the present study, T-DCQT and M-DCQT treatment restored the increased NF-*κ*B and TLR4 proteins expressions in the ileum following intestinal manipulation. These results suggest that inhibition of intestinal motility after operation in the mice is at least partially dependent on modulation of NF-*κ*B and TLR4 signaling pathways and then the inflammatory responses. However, it should be noted that the inhibitory effect of M-DCQT on the NF-*κ*B signaling pathway following intestinal manipulation appeared to be less potent than that of T-DCQT. Wehner et al. has reported that inhibition of p38 MAPK phosphorylation reduces the inflammatory response following surgery and prevents POI [28]. Our study indicates that administration of T-DCQT reduced p38 MAPK expression, so the inhibition of p38 MAPK and the inflammatory responses might be the rationale of T-DCQT for restoring intestinal motility and GI transit following intestinal manipulation.

Consistent with earlier findings, we observed a significant increase in the levels of proinflammatory cytokines in the blood of POI animals [10, 29]. These inflammatory mediators contribute to the decreased GI motility associated with POI through various mechanisms such as direct cytotoxic effects and induction of nitric oxide (NO) and proteinoid production [30]. Thus, an intestinal and systemic inflammatory response may be responsible for diminished GI transit rates observed with development of POI following intestinal manipulation in mice. Agents that suppress the development of intestinal inflammation are likely to be effective in the treatment of POI. In this regard, this study provided evidence that administration of T-DCQT or M-DCQT via an enema could effectively prevent induction of the inflammatory response observed in a POI mouse model. And the inhibition of the POI-associated inflammatory response was mediated through suppression of the inflammatory signal pathways and inflammatory mediators, such as IL-1*α*, IL-6, MCP-1, MIP-1*β*, and IL-17. This anti-inflammatory effect of T-DCQT and M-DCQT likely helps to ameliorate the impaired GI motility. Furthermore, we observed that M-DCQT treatment seems to be more effective than T-DCQT treatment in modulating the levels of inflammatory cytokines in POI. But we cannot draw a conclusion that M-DCQT exerts a better role than T-DCQT in treating POI based on this research.

## 5. Conclusion

Collectively, our findings indicate that administration of T-DCQT or M-DCOT enema could ameliorate the POI-associated inflammatory response and improve GI motility in this POI mouse model, suggesting that T-DCQT or M-DCQT treatment may be a promising strategy for prophylaxis of postoperative ileus. We also find that the effect of T-DCQT and M-DCQT on the experimental POI was comparable, although M-DCQT treatment seems more effective than T-DCQT treatment on the control of some inflammatory cytokines released after intestinal manipulation, and T-DCQT is more powerful in modulating the expression of some inflammatory signal pathways in POI. Further studies are needed to understand the discrepancy and mechanism in efficacy between T-DCQT and M-DCQT.

## Figures and Tables

**Table 1:** Primers used in qPCR.

Gene	5′primer	3′primer
NF-*κ*Bp65	5′-ACTGCCGGGATGGCTACTAT-3′	5′-TCTGGATTCGCTGGCTAATGG-3′
P38	5′-TGCTTACCCTTCACCTCAGTG-3′	5′-CAAACACATCCGTGCTCTG-3′

**Table 2:** Detailed information on the composition of T-DCQT and M-DCQT with English names.

T-DCQT	12 g of *Rheum palmatum* L.	15 g of *Magnolia officinalis* Rehd. et Wils.	12 g of *Citrus aurantium* L.	9 g of *Natrii Sulfas*
M-DCQT	12 g of *Rheum palmatum* L.	15 g of *Magnolia officinalis* Rehd. et Wils.	12 g of *Citrus aurantium L.*	9 g of *Natrii sulfas*
	12 g of Chinese angelica	12 g of Ginseng	12 g of *Rhizoma Atractylodis macrocephalae*	12 g of *Radix Paeoniae Alba*

## Data Availability

The data used to support the findings of this study are available from the corresponding author upon request.

## Abbreviations

DCQT: Da-Cheng-Qi-Tang
GI: Gastrointestinal
POI: Postoperative ileus
T-DCQT: Traditional Chinese medicine with the formula DCQT
M-DCQT: Modified DCQT
NF-*κ*B: Nuclear factor kappa-B
MAKP: Mitogen-activated protein kinase
TLR: Toll-like receptors
PCR: Polymerase chain reaction
IL-1*α*: Interleukin-1*α*
IL-4: Interleukin-4
IL-6: Interleukin- 6
IL-10: Interleukin-10
IL-13: Interleukin-13
IL-17: Interleukin-17
INF-*γ*: Interferon-*γ*
TNF-*α*: Tumor necrosis factor-*α*
MCP-1: Monocyte chemotactic protein 1
MIP-1*α*: Macrophage inflammatory protein-1*α*
MIP-1*β*: Macrophage inflammatory protein-1*β*
ENS: Enteric nervous system
ACh: Acetylcholine
SP: Substance P
VIP: Vasoactive intestinal peptide
NOS: Nitric oxide synthase.
